# Central and Peripheral Changes in FOS Expression in Schizophrenia Based on Genome-Wide Gene Expression

**DOI:** 10.3389/fgene.2019.00232

**Published:** 2019-03-22

**Authors:** Jing Huang, Fangkun Liu, Bolun Wang, Hui Tang, Ziwei Teng, Lehua Li, Yan Qiu, Haishan Wu, Jindong Chen

**Affiliations:** ^1^Department of Psychiatry, The Second Xiangya Hospital, Central South University, Changsha, China; ^2^Mental Health Institute of the Second Xiangya Hospital, Central South University, Chinese National Clinical Research Center for Mental Disorders (Xiangya), Chinese National Technology Institute on Mental Disorders, Hunan Key Laboratory of Psychiatry and Mental Health, Changsha, China; ^3^Department of Neurosurgery, Xiangya Hospital, Central South University, Changsha, China; ^4^Department of Orthopedics, The Second Xiangya Hospital, Central South University, Changsha, China

**Keywords:** schizophrenia, GEO, re-analysis, FOS, amphetamine addiction pathway

## Abstract

Schizophrenia is a chronic, debilitating neuropsychiatric disorder. Multiple transcriptomic gene expression profiling analysis has been used to identify schizophrenia-associated genes, unravel disease-associated biomarkers, and predict clinical outcomes. We aimed to identify gene expression regulation, underlying pathways, and their roles in schizophrenia pathogenesis. We searched the Gene Expression Omnibus (GEO) database for microarray studies of fibroblasts, lymphoblasts, and post-mortem brains of schizophrenia patients. Our analysis demonstrated high FOS expression in non-neural peripheral samples and low FOS expression in brain tissues of schizophrenia patients compared with healthy controls. FOS exhibited predictive value for schizophrenia patients in these datasets. Kyoto Encyclopedia of Genes and Genomes (KEGG) enrichment analysis revealed that “amphetamine addiction” was among the top 10 significantly enriched KEGG pathways. FOS and FOSB, which are implicated in the amphetamine addiction pathway, were up-regulated in schizophrenia fibroblast samples. Protein–protein interaction (PPI) network analysis revealed that proteins closely interacting with FOS-encoded protein were also involved in the amphetamine addiction pathway. Pearson correlation test indicated that FOS showed positive correlation with genes in the amphetamine pathway. The results revealed that FOS was acceptable as a biomarker for schizophrenia and may be involved in schizophrenia pathogenesis.

## Introduction

Schizophrenia is a chronic and debilitating neuropsychiatric disorder affecting 1% of the population, posing a severe social and economic burden on societies worldwide (McGrath et al., 2008; Howes and Murray, 2014). Common symptoms include positive and negative symptoms and cognitive deficits (Lewis et al., 2012). Besides its complex symptomatology, schizophrenia is considered a neurodevelopmental disorder with heterogeneous, polygenic, and highly heritable etiology (Quadrato et al., 2016). Schizophrenia affects gross architectural structures, specific cell types, and ion channels across different brain regions, including the prefrontal cortex, thalamus, thalamic reticular nucleus, and basal ganglia (Heyes et al., 2015; Mouchlianitis et al., 2016; Goff et al., 2017). However, there is heterogeneity in the molecular and genetic phenotypes of patients. The precise etiology and pathogenesis underlying schizophrenia are not fully known. Identifications of gene changes as biomarkers for schizophrenia may be helpful for diagnostic assessment of patients.

Strong evidence suggests that dysfunction of multiple neurotransmitter systems may contribute to the pathophysiology of schizophrenia, including dopamine (DA), glutamate, and serotonin neurotransmission (de Bartolomeis et al., 2014). For example, *N*-methyl-D-aspartate (NMDA) receptor antagonists such as phencyclidine (PCP), ketamine, and MK-801 have psychotomimetic effects and have been used to generate pharmacological animal models of schizophrenia (Zuo et al., 2009). Another well-established pre-clinical schizophrenia rodent model is based on amphetamine (AMPH)-induced dopaminergic dysregulation (Renard et al., 2016). AMPH-induced schizophrenia-like sensorimotor cognitive deficits are severely disrupted in schizophrenia (Pedrazzi et al., 2015; Renard et al., 2016). Genes involved in these pathways have been examined to better understand the pathogenesis of schizophrenia and points to new targets for therapeutic investigation (Karam et al., 2010; Hall et al., 2015; Network et al., 2015). Environmental risk factors and their interactions with gene expression also play important roles in schizophrenia pathophysiology (Van Winkel et al., 2010). Understanding the affected pathways and specific gene expression profiles in the pathogenesis of schizophrenia may help to uncover disease-associated biomarkers for risk assessment, regulatory mechanisms, and personalization of treatment.

The recently developed technique of gene expression profiling analysis of the whole transcriptome has been widely used to identify schizophrenia-associated genes, unravel disease-associated biomarkers, and predict clinical outcomes. Several whole-genome expression studies have utilized gene expression data from human fibroblasts, blood, and post-mortem brain samples to identify gene alterations in schizophrenia patients compared with healthy controls (Rollins et al., 2010; Cattane et al., 2015). Expression studies on post-mortem brains and peripheral cells demonstrated overlapping gene expression results; however, contrasting results have also been observed in different studies. The heterogeneity between gene expression profiles in peripheral cells and in post-mortem brains can be attributed to intrinsic differences in expression levels between the central nervous system (CNS) and peripheral tissues; and confounding factors in patients themselves, including the course of disease, age, living habits, environmental events, and clinical medications. These problems can be partly solved by integrated analysis of gene expression data from multiple studies and multiple tissues.

On this basis, the aim of our study was to elucidate gene expression changes in the pathogenesis of schizophrenia and to acquire new potential biomarkers for diagnostic prediction. We searched for microarray data for schizophrenia from the Gene Expression Omnibus (GEO) database. Different gene expression microarray studies with schizophrenia samples were selected and compared to perform a reliable genome-wide gene expression profiling analysis. First, we analyzed different microarray gene expression studies with a sample of fibroblasts from schizophrenia patients and controls to identify differentially expressed genes. Second, we compared the gene alteration results observed in different schizophrenia post-mortem brain expression studies. Third, we compared the results by analyzing gene expression changes in samples from schizophrenia mice models. Our analysis enables identification of gene expression regulation in different body areas of schizophrenia patients, disease progression, and confirmation of pre-clinical studies. This study may help us identify risk genes for schizophrenia and the underlying pathways. Our study may be applicable in elucidating the pathogenesis of other neuropsychiatric disorders.

## Materials and Methods

### Inclusion Criteria

To identify specific gene expression changes in schizophrenia, we performed a systematic search in the GEO database for schizophrenia studies. The keywords used for the GEO database search were: “schizophrenia” AND “expression profiling by array” for “study type” AND “tissue” for “attribute name” AND “homo sapiens” for “organism.” Details of the search strategy and analysis process are outlined in Supplementary Figure S1. The final studies were selected based on the criteria that (1) transcriptomic profiles were analyzed in skin fibroblasts of schizophrenia patients; (2) original data in CEL format could be downloaded; and (3) matched samples of healthy controls were used.

After screening 26 studies in GEO, the GEO dataset GSE62333^1^ was selected, which studied 20 schizophrenia patients matched with 20 healthy controls with human skin fibroblast samples based on the GPL11532 [HuGene-1_1-st] Affymetrix Human Gene 1.1 ST Array (Cattane et al., 2015). Array GPL11532 has 33,297 probes. All schizophrenia patients fulfilled the DSM-IV criteria for schizophrenia. The control samples consisted of healthy volunteers without drug or alcohol abuse and without family history of psychiatric diseases. Other diseases such as hypothyroidism or hyperthyroidism, metabolic disorders, and serious illnesses were also excluded in both groups.

To maintain consistency, we also selected GEO datasets with lymphoblast samples from schizophrenia patients. Finally, dataset GSE73129^2^, which is based on platform GPL570 [HG-U133_Plus_2] Affymetrix Human Genome U133 Plus 2.0 Array was selected for validation. Array GPL570 contained 54,675 probes. Human lymphoblast samples from 36 schizophrenia patients and 41 healthy controls were included. We compared gene expression levels in human prefrontal cortex samples in GSE92538^3^, comprising 45 schizophrenia patients and 46 healthy controls. We also validated the results with a genome-wide transcriptomics analysis of mouse prefrontal cortex and hippocampus samples. GSE10784^4^ was selected, which contained 10 Df(16)A/+ mice and 10 control mice.

### Data Analysis

Gene Expression Omnibus^5^ provides raw data (CEL file) and normalized data downloads of the: (1) citation information, (2) geo-annotation, and (3) statistical matrix. Four GEO datasets were included in the analysis, GSE62333, GSE73129, GSE92538, and GSE10784. All of the original data came from publicly available datasets.

R language was mainly used for statistical analysis. The original CEL data was subjected to background correction, normalization, and expression calculation before further analysis. We completed the data preprocessing by RMA (R package “affy”) (Gautier et al., 2004). Probes that did not match any known genes were removed. Only probes with the highest interquartile range were included for further analysis, if more than one probe matched with a gene. The selected probes were subjected to annotation using the R annotation the package “annotate.” The gene expression profiling data was transformed by log_2_ and extracted for DEG identification and analysis. The DEGs were identified using the R package “limma.” Spearman correlation analysis was performed using the “circlize” package (Gu et al., 2014), ROC curves were derived using the “pROC” package (Robin et al., 2011). Principal component analysis (PCA) was performed using the prcomp function of R “stats” package, and visualization was done using the ggplot2 package. The heatmap of DEGs was generated by clustering (using the R package “pheatmap”) with *p*-value <0.05 between schizophrenia and control samples. Associated KEGG (Kyoto Encyclopedia of Genes and Genomes) enrichment analysis of the 250 DEGs was generated by Omics Bean and illustrates the results of KEGG pathway analysis. STRING database (version 9.1) was used to analyze protein–protein interaction (PPI) networks of the candidate genes. Dot plots of expression levels of identified genes were drawn by GraphPad Prism. All statistical tests were two-sided. A *p*-value less than 0.05 is considered to be statistically significant.

## Results

### Identification and Functional Analysis of Differentially Expressed Genes (DEGs) in Schizophrenia and Healthy Control

**FIGURE 1:**
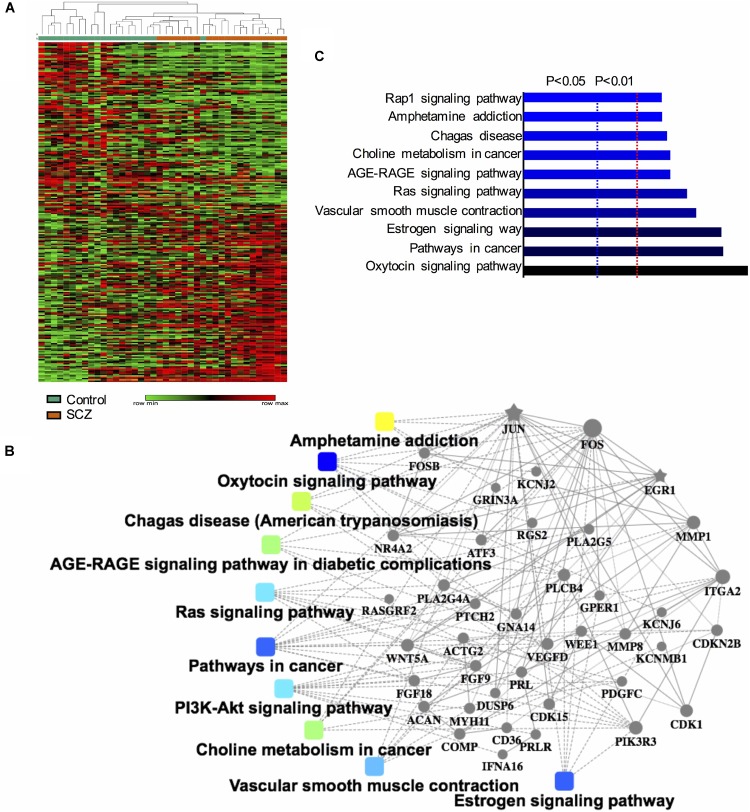
Identification and functional analysis of 250 differentially expressed genes (DEGs) between schizophrenia and healthy control samples in GEO dataset GSE62333. **(A)** Heatmap showing 250 DEGs (| FC| > 1.3, *p*-value < 0.05) between schizophrenia and control samples. Each column represents the expression level of a gene, and each row represents a sample. The color scale below the heatmap represents the raw Z-score ranging from green to red (low to high expression level). Dendrograms above correspond to the Pearson correlation-based hierarchical clustering of the 250 genes. **(B)** Associated KEGG signaling pathways of the 250 DEGs. The network model generated by Omics Bean illustrates the results of KEGG pathway and biological process enrichment analysis. **(C)** A histogram according to the KEGG functional analysis displaying the percentage of genes affected in these pathways.

The GEO dataset GSE62333 was selected to identify DEGs in human skin fibroblast samples from schizophrenia patients and matched healthy controls. After the samples were normalized by the robust multichip averaging (RMA) process, the expression levels (transformed by log_2_) of all included samples were extracted. The DEGs between two groups were calculated by the Classical Bayesian algorithm with 33,297 probes included. After screening using the criteria of | FC| > 1.2 and *p*-value < 0.05, 1,022 probes with significantly different expression levels between the two groups (517 up-regulated probes and 505 down-regulated probes) were identified. Probes that did not match any known genes were removed in further analysis. We selected the 250 probes with the highest inter-quartile range (125 up-regulated probes and 125 down-regulated probes, respectively). A heatmap that shows the 250 top-regulated genes matched with 250 probes between schizophrenia and control groups is presented in Figure 1A. The hierarchical clustering of these 250 genes demonstrated significant differences between schizophrenia and control groups.

We performed KEGG enrichment analysis of the 250 DEGs for functional analysis to identify the biological processes, pathways, and networks shared by these genes. The top 10 significantly enriched KEGG pathways (*p*-value < 0.05) were “AMPH addiction,” “oxytocin signaling,” “pathways in cancer,” “estrogen signaling,” “vascular smooth muscle contraction,” “Ras signaling,” “AGE-RAGE signaling pathway in diabetic complications,” “choline metabolism in cancer,” “Chagas disease,” and “Rap1 signaling.” The network model generated by Omics Bean illustrates the results of KEGG pathway enrichment analysis (Figure 1B). A histogram was plotted based on the functional analysis to display the percentage of genes affected in these pathways (Figure 1C).

### *FOS* Up-Regulation in Schizophrenia Peripheral Samples

**FIGURE 2:**
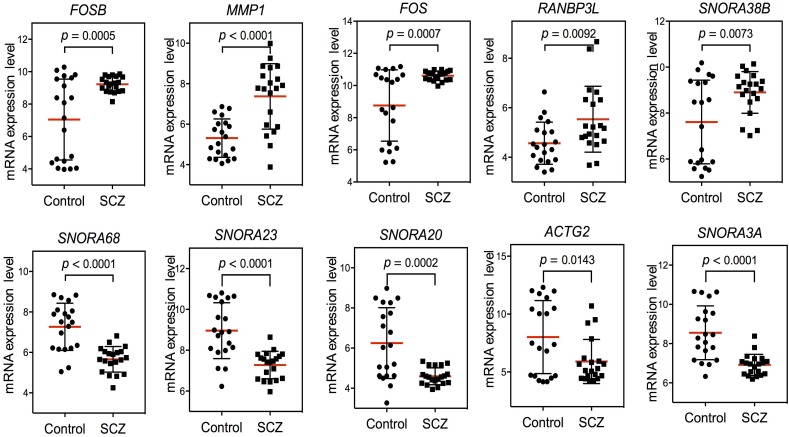
Scatter plot of expression levels of the identified top 10 differentially expressed genes (DEGs) in GEO dataset GSE62333. The top five up-regulated genes (*FOSB*, *MMP1*, *FOS*, *RANBP3L*, and *SNORA38B*) and top five down-regulated genes (*SNORA68*, *SNORA23*, *SNORA20*, *ACTG2*, and *SNORA3A*) were arranged by degree of change. Detailed information on these genes is listed in Table 1.

After confirming the pathways of 250 DEGs by KEGG enrichment analysis, we ranked the top 10 DEGs between the schizophrenia and control samples to determine which pathways these genes were involved in. The top five up-regulated genes (*FOSB, MMP1, FOS, RANBP3L*, and *SNORA38B*) and top five down-regulated genes (*SNORA68, SNORA23, SNORA20, ACTG2*, and *SNORA3A*) were arranged by degree of change. Detailed information on these genes is listed in Table 1. Dot plots illustrated that all 10 DEGs were differentially expressed between the two groups (*p*-value < 0.05), suggesting that these genes may be useful as biomarkers of schizophrenia (Figure 2). We also performed PCA and while there was a separation of some samples into diagnostic groups, overall there was no clear separation between control and SCZ samples (Supplementary Figure S2).

**Table 1:** Top 10 differentially expressed genes, either up-regulated or down-regulated.

Gene symbol	Gene	Category	logFC	Fold-change	*P*-value
*FOSB*	FosB proto-oncogene, AP-1 transcription factor subunit	Protein coding	2.28	4.8567795	0.0005
*MMP1*	Matrix metallopeptidase 1	Protein coding	1.99	3.97237	<0.0001
*FOS*	Fos proto-oncogene, AP-1 transcription factor subunit	Protein coding	1.96	3.8906198	0.0007
*SNORA38B*	Small nucleolar RNA, H/ACA box 38B	RNA	1.37	2.5847057	0.0073
*RANBP3L*	RAN binding protein 3 like	Protein coding	1.27	2.4116157	0.0092
*SNORA68*	Small nucleolar RNA, H/ACA box 68	RNA	−1.64	−3.11665832	<0.0001
*SNORA23*	Small nucleolar RNA, H/ACA box 23	RNA	−1.63	−3.09512999	<0.0001
*SNORA20*	Small nucleolar RNA, H/ACA box 20	RNA	−1.62	−3.07375036	0.0002
*ACTG2*	Actin, gamma 2, smooth muscle, enteric	Protein coding	−1.59	−3.01049349	0.0143
*SNORA3A*	Small nucleolar RNA, H/ACA box 3A	Protein coding	−1.58	−2.9896985	< 0.0001

Among the top 10 DEGs, five of them (*SNORA38B, SNORA68, SNORA23, SNORA20*, and *SNORA3A*) were non-coding genes. Five of the top 10 DEGs were protein-coding genes (*RANBP3L, ACTG2, MMP1, FOSB*, and *FOS*). Two genes in the *FOS* family (*FOS* and *FOSB*) were up-regulated in schizophrenia samples. Among the top 10 DEGs, *FOS* was most closely related to schizophrenia pathogenesis. Previous research has shown that NMDA receptor antagonist-induced and AMPH-induced pharmacological animal models of schizophrenia can up-regulate *FOS* gene expression (Zuo et al., 2009; Renard et al., 2016). And we have observed that “AMPH addiction” was one of the most significantly enriched KEGG pathways in schizophrenia samples. This suggested that *FOS* may have some connection with AMPH addiction pathway in schizophrenia samples.

**FIGURE 3:**
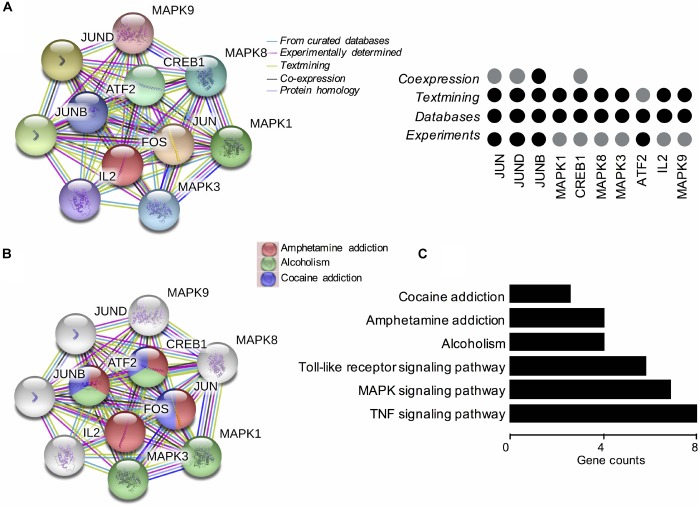
Functional protein–protein network analysis of *FOS* gene using STRING database. **(A)** The protein–protein interaction network of FOS and 10 other proteins. Proteins are presented by network nodes of different colors. The edges between FOS and its predicted functional partners represent protein–protein associations. Categorized identification of protein–protein interactions using different methods are labeled by circles. Already known interactions can be found from curated databases or experimental results. Predicted interactions can come from text mining, co-expression, and protein homology. **(B)** Further functional enrichment analysis of the protein network in KEGG pathways. FOS, JUN, CREB1, and ATF2 are involved in the amphetamine pathway. Proteins closely interacting with FOS protein also participate in other psychoactive drug pathways, such as cocaine addiction and alcoholism. **(C)** KEGG functional enrichment analysis of all genes in the protein–protein interaction network.

Based on these findings, we constructed PPI networks of FOS to explore the known and predicted proteins interacted with FOS, as well as the underlying pathways using STRING. The STRING database is a biological database and web resource which can be used for exploring the known and predicted interaction networks of a particular protein. Ten gene-encoded proteins (JUN, CREB1, ATF2, JUNB, JUND, MAPK1, MAPK3, MAPK8, MAPK9, and IL2) were in the interaction network of FOS (Figure 3A). The edges between FOS and its 10 predicted functional partners represent protein–protein associations. The edges between the proteins do not necessarily imply binding interactions; edges in different colors point to different methods of identifying interactions. Already-known interactions can be found from curated databases or experimental results. Predicted interactions arise from text mining, co-expression, and protein homology. All proteins interacting with FOS were generated from known results from curated databases and experiments. Several interactions were predicted by text mining or co-expression. Further functional enrichment analysis of the protein network in KEGG pathways revealed that FOS protein is involved in the AMPH pathway. Three proteins that interact with FOS protein (JUN, CREB1, and ATF2) were also implicated in the AMPH pathway (Figure 3B). Proteins closely interacting with FOS protein were also found to participate in other psychoactive drug pathways, such as cocaine addiction and alcoholism. KEGG functional enrichment analysis of all genes in the network is shown in Figure 3C.

**FIGURE 4:**
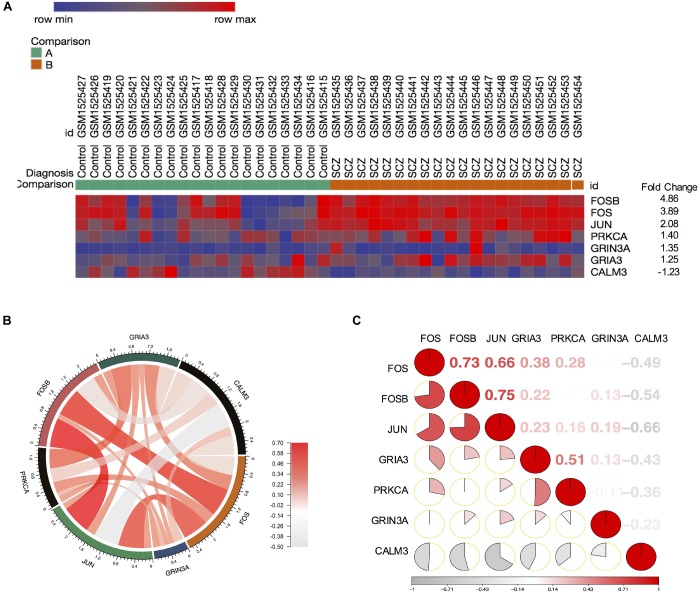
*FOS* was positively correlated with *FOSB*, *JUN*, and *GRIA3* in the amphetamine pathway in GEO dataset GSE62333. Spearman correlation of related gene expression levels of fold changes were calculated for further analysis. **(A)** Heatmap showing genes involved in the amphetamine pathway from 1,022 probes with significantly different gene expression levels (using the R package “pheatmap”). **(B)** Correlation between *FOS* and other genes in the amphetamine pathway (*FOSB*, *JUN*, *PRKCA*, *GRIA3*, *GRIN3A* and *CALM3*). **(C)** Corrgram shows the correlation of *FOS* with other six genes in the amphetamine pathway. The cells of the matrix were shaded to show the correlation value. The correlation *r*-value between FOS and six genes in the amphetamine pathway were *FOSB* (*r* = 0.73, *p* = 1.02E-07), *JUN* (*r* = 0.66, *p* = 0.000003) *GRIA3* (*r* = 0.38, *p* = 0.014374), *PRKCA* (*r* = 0.28, *p* = 0.001441), *GRIN3A* (*r* = –0.006, *p* = 0.966528), *CALM3* (*r* = –0.49, *p* = 0.001441), respectively.

Our finding from PPI networks of FOS suggested FOS is tightly related to genes involved in FOS. Therefore, we further explicate the roles of the *FOS* gene and the AMPH pathway in the pathogenesis of schizophrenia. We calculated genes involved in the AMPH pathway from the 1,022 probes with significantly different gene expression levels (using the R package “pheatmap”). Seven genes in the AMPH pathway are shown in the heatmap (Figure 4A). To examine the relationship between *FOS* and other genes in the AMPH pathway, we calculated a Spearman correlation of gene expression levels of fold changes using the GSE62333 dataset. The results indicated that *FOS* was positively correlated with other genes in the AMPH pathway, especially *FOSB, JUN, and GRIA3* (Figure 4B). Corrgram is a visual display technique to represent the pattern of correlations. To get further understanding the relationship between *FOS* gene and the AMPH pathway, Corrgram was derived according to *r*-value between *FOS* and six genes in the AMPH pathway (Figure 4C). There was a positive correlation between *FOS* and the following genes: *FOSB* (*r* = 0.73, *p* = 1.02E-07), *JUN* (*r* = 0.66, *p* = 0.000003) *GRIA3* (*r* = 0.38, *p* = 0.014374). There was a negative correlation between *FOS* and *CALM3* (*r* = −0.49, *p* = 0.001441). There was no correlation between FOS and the following genes: *PRKCA* (*r* = 0.28, *p* = 0.076489), *GRIN3A* (*r* = −0.006, *p* = 0.966528).

**FIGURE 5:**
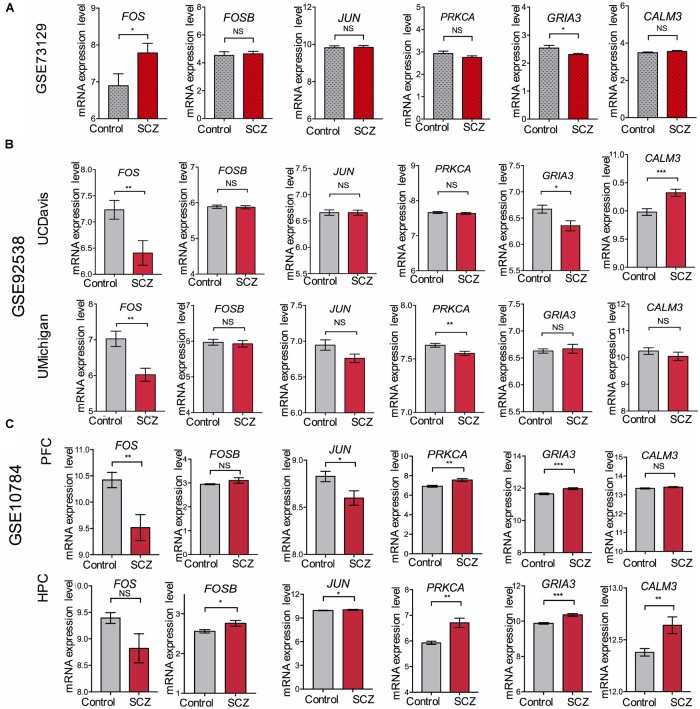
Validation of expression levels of *FOS* and other genes involved in the amphetamine pathway in GEO datasets GSE73129, GSE92538, and GSE10784. The expression of *FOS*, *FOSB*, *JUN*, *PRKCA*, *GRIA3*, and *CALM3* between schizophrenia and control lymphoblast samples have been presented in the histograms. MRNA expression level means the intensity of the detected fluorescence intensity transformed by log_2_. NS indicates not significant, ^∗^ indicates *p*-value < 0.05, ^∗∗^ indicates *p*-value < 0.01, ^∗∗∗^indicates *p*-value < 0.001. **(A)** Expression levels of *FOS* and other genes involved in the amphetamine pathway between schizophrenia and control lymphoblast samples in GEO dataset GSE73129. **(B)** Expression levels of *FOS* and other genes involved in the amphetamine pathway in human post-mortem dorsolateral prefrontal cortex samples from UCDavis or UMichigan. Samples were collected from schizophrenia patients and healthy controls. **(C)** *FOS* and other genes involved in the amphetamine pathway expression changes in the prefrontal cortex (PFC) and hippocampus (HPC) of the Df(16)A+/– schizophrenia mouse model.

**FIGURE 6:**
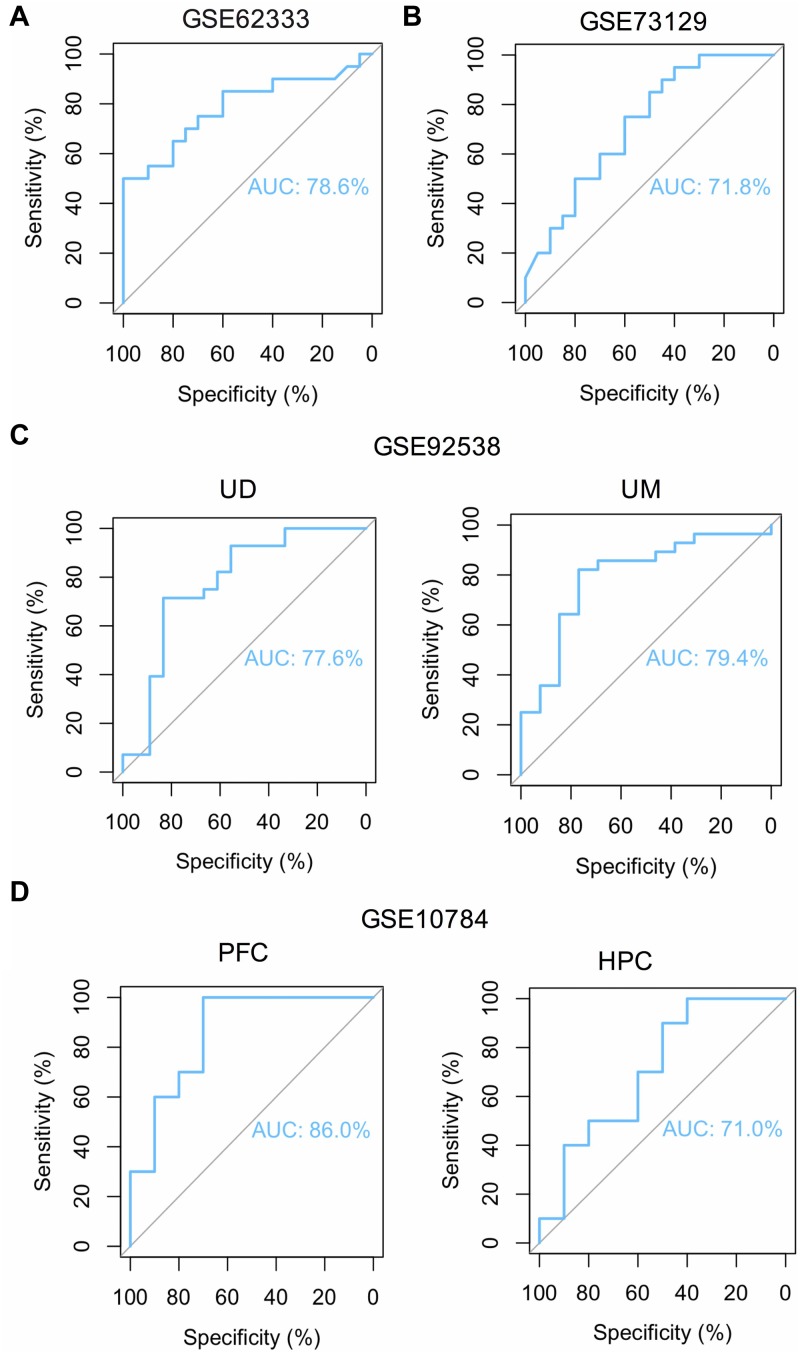
ROC curve analysis reveals the predictive value of *FOS* for schizophrenia patients in different GEO datasets. Area under the curve (AUC) is measured in order to determine the predictive value of the used datasets. **(A)** ROC curve of *FOS* in GEO dataset GSE62333 which analyzed lymphoblast samples from schizophrenia patients and controls (*P* < 0.05). **(B)** ROC curve of *FOS* in GEO dataset GSE73129 which analyzed lymphoblast samples from schizophrenia patients and controls (*P* < 0.05). **(C)** ROC curve of *FOS* in GEO dataset GSE92538 which analyzed human post-mortem dorsolateral prefrontal cortex samples from UCDavis (UD) or UMichigan (UM). Samples were collected from schizophrenia patients and healthy controls (*P* < 0.05). **(D)** ROC curve of *FOS* in GEO dataset GSE10784 which analyzed the prefrontal cortex (PFC) and hippocampus (HPC) samples from the Df(16)A+/– schizophrenia mouse model (*P* < 0.05).

To overcome the limitations of individual studies, we validated our findings in dataset GSE73129. The results showed a significant difference of *FOS* expression between schizophrenia and control lymphoblast samples. No differences were found in other genes involved in the AMPH pathway (Figure 5A). The results from two GEO datasets GSE62333 and GSE73129 demonstrated up-regulated expression of *FOS* in fibroblast and lymphoblast samples from schizophrenia patients, which were involved in the AMPH pathway. These non-neural peripheral cells may be useful for studying molecular signatures in psychiatric disorders (Cattane et al., 2015; Horiuchi et al., 2016). Post-mortem brains are also valuable tools to identify molecular alterations in these diseases. To further address the role of *FOS* in schizophrenia pathogenesis, we analyzed human postmortem dorsolateral prefrontal cortex samples and brain tissues of a schizophrenia 22q11-deletion mouse model (Stark et al., 2008; Hagenauer et al., 2016). *FOS* gene expression was significantly down-regulated in CNS tissues, regardless of the source of postmortem tissue (UCDavis or UMichigan) or the region of mouse brain (prefrontal cortex or hippocampus) (Figure 5B,C). To measure the predictive value of *FOS* in the datasets we used, ROC curves for FOS expression in schizophrenia samples and control samples were performed. The area under the curve (AUC) is 78.6% and 71.8% in GSE62333 and GSE73129 dataset, respectively. The AUC in GSE92538 is 77.6% (UCDavis) and 79.4% (UMichigan), respectively. The AUC in GSE10784 is 86.0% (prefrontal cortex) and 71.0% (hippocampus), respectively. The highest AUC was observed in the prefrontal cortex samples of GSE10784 dataset. These results suggested that *FOS* is acceptable as a biomarker of schizophrenia. Details were presented in Figure 6.

Collectively, our findings revealed that *FOS*, which is involved in the AMPH pathway, is significantly up-regulated in human fibroblast samples and can be validated in peripheral lymphoblast samples. Further analysis showed contrasting expression of *FOS* in CNS tissues. *FOS* was down-regulated in human postmortem dorsolateral prefrontal cortex samples and brain tissues of a schizophrenia 22q11-deletion mouse model.

## Discussion

In this study, we performed a systematic search on the GEO database for microarray studies of schizophrenia with samples from patients’ fibroblasts, lymphoblasts, post-mortem human brains, and brains from a mouse model of schizophrenia. Unlike previous studies, which analyzed peripheral or CNS samples only from schizophrenia patients, we identified *FOS* expression changes in different tissues. Skin fibroblasts, lymphocytes, and lymphoblasts are frequently used in schizophrenia gene expression analysis because of the difficulty in obtaining CNS tissues or cells from schizophrenia patients. Postmortem brains are another good source that help to address this problem. Our study, which analyzed *FOS* gene expression in peripheral and CNS tissues, provides a new window into the molecular changes underlying schizophrenia pathogenesis.

In our analysis, we re-analyzed publicly available microarray gene expression data and obtained different conclusions. A previous study of GEO datasets GSE62333 also reported the up-regulation of *FOSB* and *FOS* in the fibroblasts of schizophrenia patients and mainly focused on *EGR1* and other genes as biomarkers for disease diagnosis (Cattane et al., 2015). Our analysis focused on gene expression changes and related pathways contributing to the pathogenesis of schizophrenia. The analysis of the top 10 DEGs in schizophrenia and healthy samples revealed that *FOS* and *FOSB* (which are involved in the AMPH addiction pathway), were significantly up-regulated in schizophrenia fibroblast samples. Protein functional network analysis revealed proteins closely interacting with FOS that were also involved in the AMPH addiction pathway. These findings were consistent with our KEGG enrichment analysis results, which listed “AMPH addiction” as one of the top 10 significantly enriched KEGG pathways. Additionally, Spearman correlation analyzing samples in dataset GSE62333 indicated that *FOS* was positively correlated with *FOSB, JUN, and GRIA3* in the AMPH pathway, and negatively correlated with *CALM3* in the AMPH pathway. Thus, our results support previous findings and reveal a previously unrecognized pathway connected with *FOS* in the pathogenesis of schizophrenia.

As one of the most studied immediate early genes in the brain, *FOS* is highly expressed in addiction to psychoactive drugs such as AMPH, alcohol, and cocaine (Gallo et al., 2018). It also has been reported to be differentially expressed in schizophrenia samples, either through experimental or expression profiling studies (Zuo et al., 2009; Cattane et al., 2015; Renard et al., 2016; Monfil et al., 2018). Besides, *FOS* is thought to play an important role in the pathophysiology of schizophrenia (Zuo et al., 2009; Renard et al., 2016; Monfil et al., 2018). We observed that *FOS* expression was highly up-regulated in schizophrenia group of dataset GSE62333 which was then validated in another dataset GSE73129. ROC curves indicated fair predictive values of the datasets we analyzed. These combined results suggested FOS is acceptable as a biomarker of schizophrenia. Detection of FOS in blood samples may be helpful for schizophrenia diagnosis. Further exploration using postmortem human brains and 22q11-deletion mouse brain samples suggested that FOS is up-regulated in non-neural peripheral samples and down-regulated in brain tissues of schizophrenia patients compared with those of healthy controls. The alteration of *FOS* expression in peripheral and CNS tissues of schizophrenia indicates that this gene is sensitive for schizophrenia.

Among the top 10 DEGs we detected, five of them (*SNORA38B, SNORA68, SNORA23, SNORA20*, and *SNORA3A*) were non-coding genes. The ratio of non-coding to protein-coding genes is considered a function of developmental complexity. Prokaryotes have less than 25% non-coding DNA, while humans have approximately 98.5% non-coding DNA. RNA-based regulation is devoted to the majority of advances in human genomic programming (Mattick, 2004). Increasing evidence suggests that non-coding RNAs play an important role in neural development and function, and neurological disease progression (Mehler and Mattick, 2006). Although non-coding RNAs such as small nucleolar RNAs (snoRNAs), microRNAs and long non-coding RNAs (LncRNAs), have been studied in disease etiology (Barry, 2014; Lai et al., 2016), the involvement of *SNORA38B*, *SNORA68, SNORA23, SNORA20*, and *SNORA3A* in the pathogenesis of schizophrenia has yet to be reported. However, snoRNAs may play important roles in brain development and neurological disease progression (Pang et al., 2006). Recent studies have indicated that snoRNAs are highly expressed in tumor cells and are involved in cell behavior and oncogenesis (Williams and Farzaneh, 2012; Gong et al., 2017; Zhou et al., 2017).

Five of the top 10 DEGs were protein-coding genes (*RANBP3L, ACTG2, MMP1, FOSB*, and *FOS*). *RANBP3L* is a gene involved in detoxification which mediates bone morphogenetic protein (BMP)-specific nuclear export of SMAD to terminate BMP signaling (Chen et al., 2015). The involvement of *RANBP3L* in schizophrenia has yet to be reported, but exon microarray analysis of human dorsolateral prefrontal cortex revealed up-regulation of this gene in alcoholism (Manzardo et al., 2014). *ACTG2* encodes gamma 2 enteric actin, which is a smooth muscle actin found in enteric tissues and is crucial for enteric muscle contraction (Thorson et al., 2014; Wangler et al., 2014; Matera et al., 2016). *ACTG2* has yet to be associated with schizophrenia. Another actin-coding gene, *ACTG1*, is associated with brain development and hearing loss (Perrin et al., 2010; Park et al., 2013). Matrix metalloproteases (MMPs) are involved in the degradation of basement membrane and extracellular matrix (ECM) components in physiological and pathological processes, such as tissue development, wound healing, and tumor invasiveness (Poola et al., 2005). As a member of the MMP family, *MMP1* encodes an enzyme that breaks down the ECM, and promotes tumor cell division and metastasis. Studies have shown that activator protein-1 (AP-1) genes *FOS* and *JUN* can regulate *MMP1* gene expression in invasive tumors (Poola et al., 2005; Uhlirova and Bohmann, 2006; Baker et al., 2018). MMP-mediated ECM disruption is also involved in the pathogenesis of schizophrenia. MMP modulators may therefore be a potential therapeutic target for the treatment of schizophrenia (Chopra et al., 2015). Besides, FOS proteins can dimerize with JUN proteins to form the transcription factor complex AP-1, which regulates cell proliferation, differentiation, and transformation (Milde-Langosch, 2005). Few studies have reported functions of *FOSB* in the CNS. However, a truncated splice variant of *FOSB* named *Delta-FOSB* is involved in behavior and addiction to drugs (Nestler, 2008; Ruffle, 2014). *FOS* was up-regulated in schizophrenia samples and is a recognized marker of neural activation (Gallo et al., 2018).

There are several limitations in our study. First, the gene expression profiles could be affected by many factors, such as differences in methodology and/or sample preparation, patient characteristics, platform, and data analysis (Sullivan et al., 2006; Mistry and Pavlidis, 2010; Kumarasinghe et al., 2012; Mistry et al., 2013). Studies on schizophrenia are often associated with small sample sizes which may result in low statistical power. Second, several studies reported that peripheral cells may not reflect the gene expression profile of the human brain. Furthermore, using postmortem brains to understand dynamic changes in disease progression and development of complications is challenging (Rollins et al., 2010; Tylee et al., 2013; Cattane et al., 2015; Horiuchi et al., 2016). Finally, confirmatory experiments and comparison of results with microarray gene expression modifications from re-analysis will enable validation of our results.

## Conclusion

To conclude, our results indicate that *FOS* was significantly up-regulated in schizophrenia fibroblast and lymphoblast samples. Exploration of *FOS* gene expression in CNS tissues revealed that this gene was largely down-regulated in datasets GSE92538 and GSE10784. The signatures we identified are consistent with current hypotheses of molecular dysfunction in schizophrenia, including alteration of the *FOS* gene and involvement of the AMPH addiction pathway. *FOS* and AMPH-related genes may thus represent novel biomarkers for diagnosis of schizophrenia in clinical practice.

## Author Contributions

JH and FL performed data extraction and prepared the manuscript. BW, HT, and ZT reviewed the literature and performed the quality assessment. YQ and LL reviewed the revised manuscript and checked the grammars. HW and JC supervised all the work.

## Conflict of Interest Statement

The authors declare that the research was conducted in the absence of any commercial or financial relationships that could be construed as a potential conflict of interest.

## Footnotes

1 https://www.ncbi.nlm.nih.gov/geo/query/acc.cgi?acc=GSE62333

2 https://www.ncbi.nlm.nih.gov/geo/query/acc.cgi?acc=GSE73129

3 https://www.ncbi.nlm.nih.gov/geo/query/acc.cgi?acc=GSE92538

4 https://www.ncbi.nlm.nih.gov/geo/query/acc.cgi?acc=GSE10784

5 https://www.ncbi.nlm.nih.gov/geo/

## Supplementary Material

FIGURE S1 Flowchart showing the identification and selection of microarray studies from Gene Expression Omnibus (GEO) and the data analysis procedure.

FIGURE S2 Transcriptomic analysis via principle component analysis including control and SCZ samples in GEO dataset GSE62333 using top 10 differentially expressed genes.
