# Supplementary figures and images for: Central and Peripheral Changes in FOS Expression in Schizophrenia Based on Genome-Wide Gene Expression
Source: Front Genet. 2019 Mar 22;10:232. doi: 10.3389/fgene.2019.00232 (PMC6439315; doi:10.3389/fgene.2019.00232)

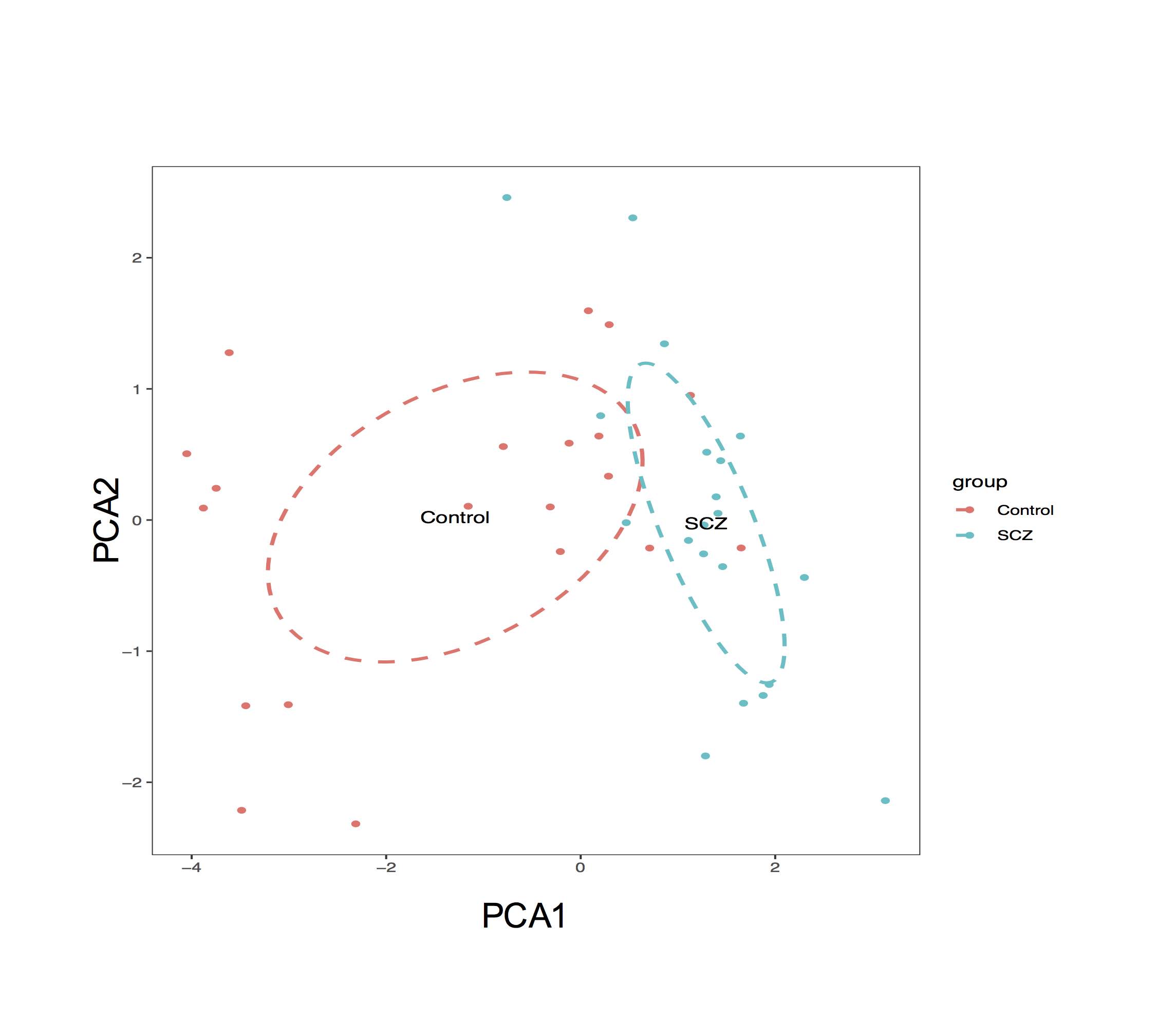

Supplement: FIGURE S2 — Transcriptomic analysis via principle component analysis including control and SCZ samples in GEO dataset GSE62333 using top 10 differentially expressed genes. [file Image_2.JPEG]
